# The role of user context in the design of mobile map applications

**DOI:** 10.1080/15230406.2021.1933595

**Published:** 2021-07-06

**Authors:** Mona Bartling, Anthony C. Robinson, Bernd Resch, Anton Eitzinger, Karl Atzmanstorfer

**Affiliations:** a Department of Geoinformatics – Z_GIS, University of Salzburg, Austria; b Department of Geography, GeoVISTA Center, the Pennsylvania State University, University Park, PA, USA; c Center for Geographic Analysis, Harvard University, Cambridge, MA, USA; d Alliance of Bioversity International and CIAT, Americas Hub, Cali, Colombia

**Keywords:** User context, user experience, map adaptations, map design, mobile cartography, map applications

## Abstract

Mobile map applications are typically used by a broad range of users. Users can be diverse in their context attributes (e.g. map use experience, activities during map use), and several previous user experience (UX) studies have focused on understanding how some contextual factors influence the UX for designing maps that satisfy users’ needs. A need for research remains to evaluate the relationship between user context, UX, and variants of mobile map element design. In this article, we present our research investigating the interplay of these factors through an empirical user study with citizens in Austria. We created an online survey and generated 84 map variations, combining seven map-related tasks, three base map styles, two map detail densities, and two time-pressure variants. We tested these map variations with 107 survey participants and related their UX to user context. Map-related tasks emerged as a dominant factor modifying the map design UX. Further results showed that interactivity loaded map-related tasks were aided when paired with low detail-dense base maps, contrasting overlay features. We recommend future research to analyze an extended set of context attributes with additional participant data, to evaluate dynamic variations in context, and to find ways to dynamically monitor mobile map design UX.

## 1. User context in designing mobile map applications

Mobile map applications are increasingly used in different aspects of our daily routines and by professional and private users alike. These applications can be used for different purposes, by users of all kinds of demographics and digital or spatial literacy, and by users of different cultures and countries. The map application design plays a crucial role in creating a positive experience for all of these users and purposes of use (Roth et al., 2017). Hence, a User-Centered Design (UCD) approach is relevant to inform the application design according to the needs of the users (Roth et al., 2015).

Typical UCD methods include usability and user experience (UX) evaluation measures. *Usability* is defined as the “extent to which a product can be used by specified users to achieve specified goals with effectiveness, efficiency, and satisfaction in a specified context of use” (Bevan, 1992). The term *User Experience*, or *UX*, extends this definition and is defined as “user’s perceptions and responses that result from the use and/or anticipated use of a system, product or service” (Bevan, 1992). Therefore, evaluating an application’s UX allows us to understand users’ behavior and satisfaction when interacting with an application, as well as how useful they perceive it to be (Roth, 2017).

However, to generate effective designs, we need to know who the users of these map applications are and understand their needs, requirements, and perceptual and cognitive limits to adjust the map design (Çöltekin et al., 2020; Roth et al., 2017; Griffin, White et al., 2017; Ottley, 2020). To understand these user characteristics, we need to look at their user context. Context is commonly defined in Human-Computer Interaction (HCI) as being any relevant information that describes the user and their situation (Dey, 2001). For mobile map applications, user context might differ, ranging from subtle to more substantial differences. For example, users may have a range of demographics, technical and spatial literacy, cognitive abilities, emotional states, and other aspects. Reichenbacher (2004) models user context by distinguishing between information on the user (e.g. their demographics, preferences, knowledge, and skills), the spatio-temporal situation of the user and the physical environment, the activity a user is carrying out, information on the system (e.g. the device), and the visualized information. In contrast, Griffin et al. (2017) distinguish between information on the user, the activity a user is carrying out, the environment, and the map itself.

As Butt and Li (2015) indicate, it is relevant to consider user context when evaluating the UX of map applications as their ideal design varies based on user context factors. Accordingly, considering users and the intersections between their context is crucial for understanding the most suitable way to design map applications (Newman et al., 2010). Griffin et al. (2017), and Griffin, Robinson et al. (2017) underline that the differences found between users in their context have an impact on the design of map applications, which should be further investigated. Several studies that further support the evaluation of user context have been conducted on map design and its usability and UX. For example, Atzmanstorfer et al. (2016) emphasize the importance of designing maps with reference to local conventions; Gottwald et al. (2016) discuss challenges faced by users with low levels of computer, internet, or map literacy, especially found in older adults; Nivala and Sarjakoski (2007) examine map designs that represent use situations, e.g. temporal and seasonal changes (summer and winter), and differences in age and nationalities; Weninger (2012) considers the relevance of examining interaction patterns; and Lokka and Çöltekin (2020) review the effects on the visual design of adults with different age and cognitive abilities.

Hence, while some users have a positive experience with a map application, others might not. Thereby, a negative UX potentially influences the long-term participation and overall motivation of users to engage with a map application (Meng & Malczewski, 2009). Hence, the “accessibility, disability, literacy, and other individual user differences” (Roth, 2019, p. 2) are relevant aspects of the inclusive design of map applications.

Although various studies have discussed the impact of user context on mobile map application design, what is still underrepresented in the literature is delineating relations between user context and the UX of specific designs for map application elements. Since the range of possible design decisions for map applications is broad, and map design elements can be combined in numerous ways (e.g. regarding the interface complexity, visual style and layout, and others (Vincent et al., 2019)), it is important to evaluate these differences and possible combinations.

In this study, we evaluate mapping UX in relation to attributes of user context for a range of different mobile map application elements through an online survey carried out in Austria. According to the suggestion that design decisions depend on the users and their context (Griffin, White et al., 2017), our primary goal is to understand which kinds of mobile map application design choices yield the best UX (in terms of task success, comfort, and confidence ratings) for users with varying contextual attributes. Many user context attributes can be related to the map design UX; however, in this study, and based on the context model of Griffin et al. (2017), we focus on information about the user (i.e. participant characteristics such as demographics or map use experience), the map use activity (i.e. the tasks that participants carry out with mobile maps), and the map itself. As such, we hypothesize that the map design UX is associated with user context and explore the following research question: Is the UX of mobile map elements modified by user context attributes?

In the following sections, we first describe our methods and results and then discuss our findings, their significance, and potential limitations of our study before concluding the article with ideas for future work.

## 2. Experiment methods

**Figure 1.**
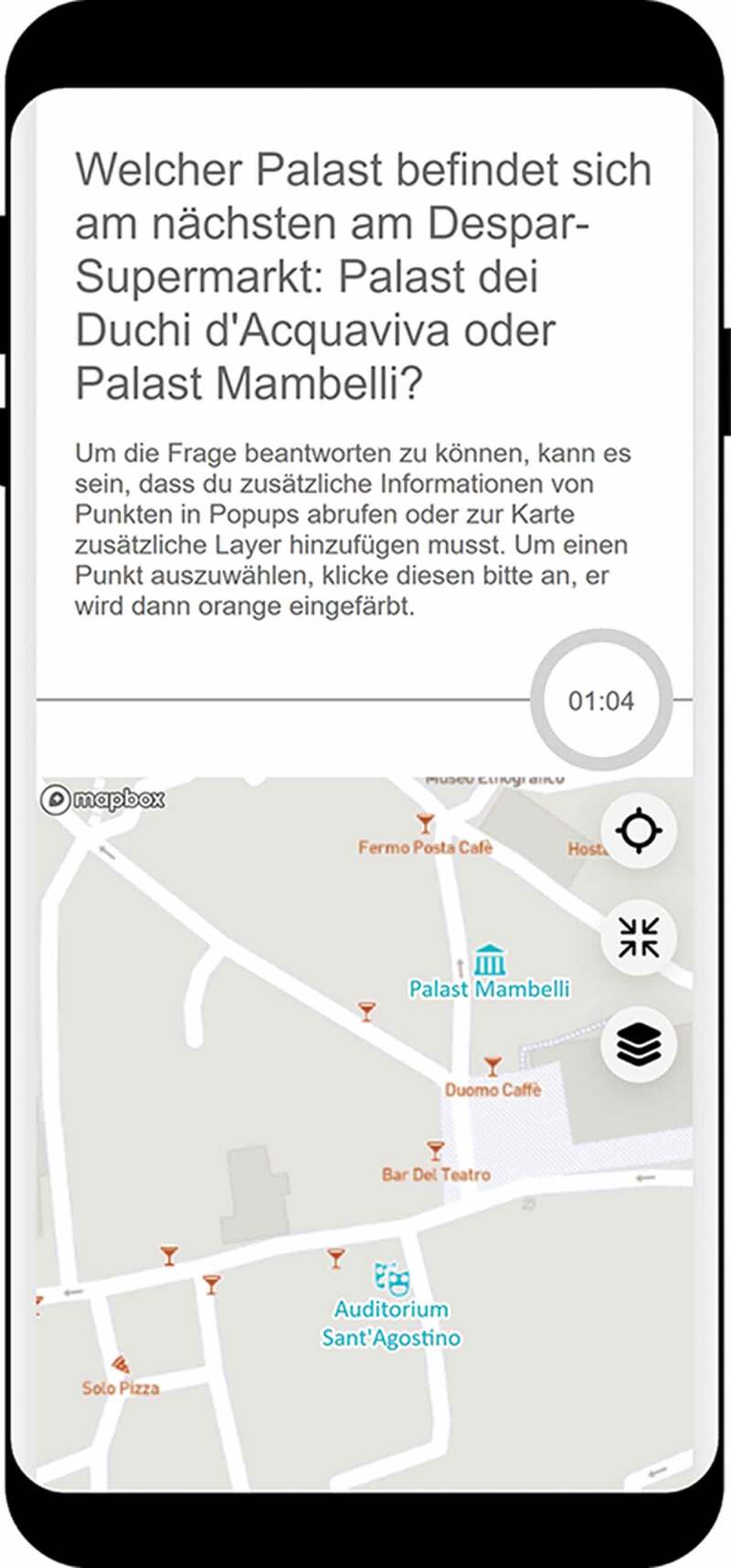
Example of a map-related task with a respective map design in the survey (note that the question corresponds to map-related task #16 of Annex in supplementary materials)

To pursue our research question and assess the potential influence of user context on map design UX, we created an online survey to test 84 map variations. We applied a between-subjects study design, where each participant was randomly assigned a subset of twelve map variations. The tested map variations consisted of seven different types of map-related tasks using different variations of map elements in the application. These map elements included a combination of three different base map styles, two map detail densities, and the map-related tasks featured two different types of time pressure. We detail these map variations in section 2.2 (see Figure 1 for an example of a map variation).

We created the survey and the map variations with the GeoCitizen application (geocitizen.org) and received responses from 107 participants from Austria. Besides collecting information on participant characteristics, we measured the task success of using each map variation and asked participants to self-report on their comfort and confidence after each map variation (see section 2.3).

We varied the map-related tasks, base map styles, and map detail densities in each subset that a participant received but did not vary the type of time pressure in the subset. Complete details describing the individual map-related tasks and map elements and how they were assigned to participants are provided in the following sections.

Initially, we had designed this study as a field survey to be carried out within the YouthMetre project (youthmetre.eu) in Atri, Italy, which uses the GeoCitizen application in their work. However, due to the COVID-19 pandemic, we were not able to carry out the experiment in Atri, so we translated it into German and distributed it as an online survey in April and May 2020 to citizens in Austria.

### 2.1. Participant recruitment

Participants were recruited through social media channels (community and “blackboard” groups on Facebook in the region of Salzburg, Austria), and we asked participants to share the survey with their relatives and friends (snowball sampling (Goodman, 1961)). We further invited students and employees from the University of Salzburg, Austria to participate and distribute the survey. We, therefore, used the weekly University newsletters and student e-mail lists.

In total, we registered 205 participants, of which 107 completed the survey. While the mean age of all participants was 35 years (Standard Deviation – SD: 14.52), the mean age of participants who completed the survey was 33.36 years (SD: 13.21). Of the 205 registered participants, 55% were women, but this number decreased to 52% for the 107 participants that completed the survey. The participants who completed the survey indicated a mean comfort rating of 4.1 for smartphone use (SD: 0.85) and 3.66 for map use (SD: 0.77). More details on the participants’ characteristics are shown in Table 1.

**Table 1.** Participant characteristics

	Total (n = 205)	Completed (n = 107)
Age	Mean: 35 SD^1^: 14.52 Min: 17 Max: 71	Mean: 33.36 SD: 13.21 Min: 18 Max: 65
Gender	Female: 113 Male: 92	Female: 56 Male: 51
How comfortable participants felt using a smartphone (1 (min) – 5 (max))	Mean: 4.03 SD: 0.84	Mean: 4.1 SD: 0.85
Whether participants have been using maps previous to the study	Yes: 198 No: 7	Yes: 104 No: 3
How comfortable participants felt using a map (1 (min) – 5 (max))	Mean: 3.56 SD: 0.86	Mean: 3.66 SD: 0.77
Map use frequency (1 (min) - 4 (max))	Mean: 2.58 SD: 0.66	Mean: 2.65 SD: 0.67
Smartphone operating system	Android: 141 iPhone: 64	Android: 70 iPhone: 37
Smartphone screen size (in inches)	Mean: 5.48 SD: 0.62	Mean: 5.35 SD: 0.6

^1^Standard Deviation.

Of the 205 registered participants, 98 participants did not complete the survey. We discuss possible reasons for this high drop-off rate in section 4.

### 2.2. Materials

We created seven different map-related tasks (Table 2), which were combined with different map element variations. These variations comprised of different base map styles, map detail densities, and types of time pressure (Table 3).

**Table 2.** Map-related tasks and required types of interactivity

Map-related tasks	Main types of interactivity to solve map-related tasks		
	Toggle overlay feature layers	Consult popup information	Visually estimate distances
T1: Create point			
T2: Select point		x	
T3: Select point (distance)	x		x (nearest/furthest POI to another POI)
T4: Select line	x	x	
T5: Select line (distance)	x		x (shortest/longest path between POIs)
T6: Select generalized polygon	x		
T7: Select detailed polygon	x

**Table 3.** Map elements

Category	Variant:
Base map style	Mapbox Dark
	Mapbox Streets
	Mapbox Satellite Streets
Map detail density	Regular (POI Mapbox pre-defined filter rank for POIs; highlights important POIs)
	Dense (no Mapbox pre-defined filter rank for POIs; provides a broader set of information, especially useful in smaller towns where Mapbox filters out important POIs)
Time pressure	Count-up timer (shows the elapsed time for each map-related task)
	Countdown clock

The seven tested *map-related tasks* were selected based on previous work (Board, 1978; Roth, 2012; Lobben, 2004) and were focused on visual searches (Liao et al., 2019). We extended the approach by Liao et al. (2019) and asked participants to engage with point, line, and polygon features. With these visual searches, constituting interactions such as identifying, comparing and interpreting, or ranking geospatial information (Roth, 2012), we asked participants to create (T1) and select features (T2-T7). These types of map-related tasks are typically found in use case scenarios of citizen participation (e.g. submit locations to build new pedestrian crossings (T1), to improve the street network (T4), or to improve parks and green areas (T6 and T7) of a city), tourism (e.g. review opening hours or other information of restaurants and museums (T2)), or mobility (e.g. compare walking distances between POIs (T3 and T5) or select panoramic routes (T4)). For all map-related tasks, participants had to interact with POI categories by evaluating their icons (e.g. schools, museums, churches, libraries). For the map-related tasks T3 to T7, the participants were required to toggle additional feature layers to find additional suitable information to solve the map-related tasks. For the map-related tasks T2 and T4, participants were further required to consult the popup information or toggle additional feature layers. For the map-related tasks T3 and T5, we asked participants to visually interpret easily detectable differences in distances between points (T3) or lines (T5). Furthermore, for the polygon feature types (T6 and T7), we distinguished between two generalization levels. For the generalized polygons (T6), we used rectangles, and, for the detailed polygons (T7), we opted for irregular shapes that represented the specific areas of the polygons in more detail.

**Figure 2.**
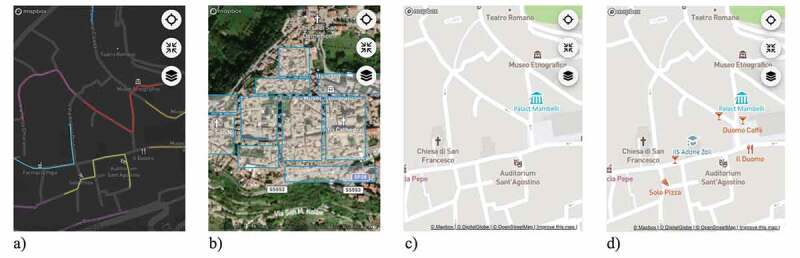
a) Dark theme base map with selectable line features of map-related tasks T4 and T5; b) Hybrid imagery base map with selectable polygon features of map-related task T6; c) Regular-density Mapbox Streets base map in combination with a selectable point feature; d) Dense Mapbox Streets base map in combination with two selectable point features

As in Konečný et al. (2011), we opted to test different types of *base map styles*. In addition to a commonly used general-purpose streets base map (Mapbox Streets, see Figure 1), we used two other Mapbox base map types for comparison: the dark theme base map (Mapbox Dark), useful to contrast selectable features on the map (Figure 2(a)), and a hybrid imagery base map (Mapbox Satellite Streets), which provides additional details of the study area (Figure 2(b)).

Following approaches by previously conducted studies (Bestgen et al., 2017; Edler et al., 2019; Liao et al., 2019; Vincent et al., 2019), the third category we varied was the *map detail density*, where we aimed to assess the impact of including more information on the mobile map UX. The area of interest of the maps was set to the village of Atri in Italy, where the filter rank for POIs – pre-defined by the Mapbox base maps – yielded a relatively low map detail density on the maps. Hence, we used the pre-defined POI density by Mapbox in a first variant (Figure 2(c)). In a second variant, we eliminated the pre-defined filter ranks, which led to base maps with a higher density of POIs (Figure 2(d)).

As in a mobile environment users can be constrained by time pressure (Meng, 2005), we aimed to analyze the map design UX when *time pressure* was applied for completing map-related tasks (Crescenzi et al., 2016; Nielsen, 2009). For this category, we used a count-up timer (counting the elapsed time) and a countdown clock. The set countdown time was different for various map-related tasks. For the map-related task of creating a point (T1), the timer was set to 60 seconds; for the rest of the map-related tasks (T2-T7), the timer was set to 90 seconds. We selected the countdown time by pretesting the map-related tasks with eight pretest participants.

In total, we created 42 different map variations, combining the map-related tasks with a respective question, base map styles, and map detail densities. We then duplicated these 42 map variations and applied a time pressure variant to each set. By combining these different category variants, we generated 84 maps in total. We applied a between-subjects experiment and participants received a survey with twelve maps using either a count-up timer or a countdown clock. The complete questions for each map-related task are included in Annex Table A1, in supplementary materials.

### 2.3. Survey structure

We divided the survey into three sections, whereby, section (1) provided instructions on how to solve the map-related tasks, section (2) contained twelve map-related tasks for the participants to solve – corresponding to different map element variations – and section (3) comprised general design preference and feedback questions. These survey sections are explained in more detail below.

#### 2.3.1. *Survey* *section 1* *: survey instructions/pretest*

We showed participants short instructional videos with relevant tips for solving the map-related tasks of the survey. In the short video snippets, we demonstrated the following interactions:

- How to move and zoom on the map.
- How to consult popup information and how to add and remove additional layers.
- How to create a point and select points, lines, and polygons.
- How to solve a map-related task and how to continue to the next map-related task.
- Where the count-up timer/countdown clock is positioned.

#### 2.3.2. *Survey* *section 2* *: map-related tasks*

Each participant was randomly assigned a set of twelve map variations (map-related tasks with respective map elements). This set of map-related tasks (with their respective map elements) was accompanied by either a count-up timer or a countdown clock. After a participant received a map-related task, we posed three follow-up questions to ascertain feedback on the participant’s comfort level and confidence when solving the map-related tasks (Table 4). With a fourth additional follow-up question (see #4 in Table 4), we aimed to pinpoint some of the specific problems the participant encountered when solving the map-related tasks. Therefore, we presented pre-defined choices for participants to choose from (multiple choice). Following our pretests, wlefte noticed several common issues, which we used to refine our prompts.

**Table 4.** Follow-up questions

#	Question	Answer type	Choices
1	Were you able to successfully solve the map-related task?	Single choice	Yes No
2	How did you feel when solving the map-related task?	Single choice	Comfortable Uncomfortable Indifferent I did not solve the map-related task
3	Do you think your solution to the map-related task was …	Single choice	Correct Incorrect I am not sure
4	Did you encounter any problems? If so, what were these?	Multiple choice	I did not understand the map-related task I was not able to find the right information Too much information was presented The colors distracted me I did not understand the symbols I had problems reading the information table of a point. I had problems turning on or off other layers Moving on the map was difficult I was not able to select or create an element on the map I did not have enough time I had other problems, such as … [free text] I did not have any problems

#### 2.3.3. *Survey* *section 3* *: base map preference questions and general remarks*

In the last survey section, we sought to compare the participants’ preferences regarding the individual base map styles. As such, we posed three different questions comparing the dark theme to the Mapbox Streets base map, the Mapbox Streets to the hybrid imagery base map, and the dark theme to the hybrid imagery base map. For each base map comparison, the participants were asked to select the base map style they liked best.

Further, we were interested in gathering general feedback, and posed the following four open-ended questions to elicit free-text responses:

- What did you like about the maps that we presented?
- What didn’t you like about the maps that we presented?
- What did you like about the map-related tasks that we presented?
- What didn’t you like about the map-related tasks that we presented?

### 2.4. Experiment procedure

The participants received the survey as an online link and completed it on their mobile devices at their convenience. The participants were invited to register their e-mail address at the end of the survey to win a 10€ Amazon voucher. In total, we prepared 20 vouchers that we sent to randomly drawn participants.

### 2.5. Data analysis

To analyze the survey results, we first calculated descriptive statistics to evaluate participants’ task success for their respective map variations. We also analyzed the comfort and confidence ratings of the participants when solving the map-related tasks.

Additionally, we applied a logistic mixed-effects regression model (Hedeker, 2003) to analyze the statistical relationships between participants’ success in completing map-related tasks and their comfort and confidence ratings, the map elements, and the individual participant characteristics. We used this type of regression model to include random effects, which was relevant in our study since we took multiple measures per participant and map variation. As such, each participant received twelve different map variations and each map variation was used by different participants. Thereby, these multiple responses per participant and map variation were not independent of each other but were hierarchical.

As we were interested in analyzing the task success, comfort, and confidence reported by our participants, we built three different models, with each metric as a binary dependent variable. For the three dependent variables, we categorized the answer types regarding participants’ completion of the map-related task as (1) successful vs unsuccessful, (2) comfortable vs uncomfortable (including participants who were neither feeling comfortable nor uncomfortable and participants who were unable to complete the map-related task), and (3) confident vs unconfident (including participants that indicated being unsure).

The models were built in R using the glmer-function of the lme4 package (github.com/lme4/lme4/). For numerical variables (e.g. age, time spent on each map variation), it was possible to evaluate the linear effect (negative/positive) between variables. For categorical variables without a particular order (e.g. map-related tasks, base map styles), we selected the variant that performed best (based on the task success, comfort, or confidence ratings) and used it as a reference level for the model. As independent variables, we inserted the remaining factors, except for the participant ID and map variation ID. The latter two variables were used as random effect variables (intercept). We also evaluated the p-value and the standardized beta coefficient (SBC). For the p-value, we assigned the statistical level of significance as 5% (p < 0.05*). We also used the SBC since it allows us to compare the coefficients to other models. The higher the value of the SBC, the more impactful the tested variable is on the dependent variable.

Lastly, we calculated descriptive statistics to evaluate the general feedback (positive and negative remarks about the map-related tasks and map variations) and the general base map preferences of the participants.

## 3. Results

In the following, we will present the results of the experiment. We first present the overall result scores of the task success, comfort, and confidence ratings and, subsequently, relate the result scores to each map variation. Lastly, we present the results of the regression analysis and participants’ general remarks and preferences. The results of our experiment are discussed and interpreted in section 4.

### 3.1. Overall task success, comfort, and confidence ratings

Each of the 84 map variations was used an average of 15.29 times. Since each map-related task (in combination with respective map elements: base map style and map detail density) was set in two versions, one with a count-up timer and the other with a countdown clock, each map-related task (with its respective map elements) was completed an average of 30.57 times. On average, participants correctly solved 65% of the map-related tasks (7.85 of 12 map-related tasks; SD: 0.21). In terms of their comfort and confidence with map-related tasks, participants reported feeling comfortable in solving 46% of the map-related tasks (5.57 of 12 map-related tasks; SD: 0.26) and confident in having correctly solved the map-related tasks in 56% of cases (6.76 of 12 map-related tasks; SD: 0.21).

**Figure 3.**
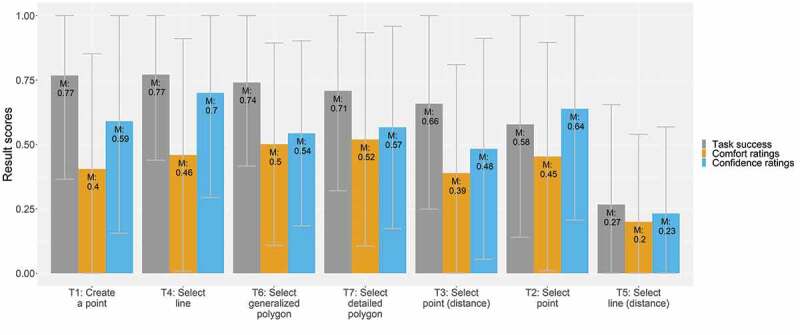
Task success, comfort, and confidence ratings by map-related tasks

Figure 3 depicts the result scores of the task success, comfort, and confidence ratings by *map-related tasks*. The highest task success (77%) was recorded for the map-related tasks of creating a point (T1) and selecting a line (T4). The lowest task success, with 27%, was recorded for the map-related task of selecting a line to identify the shortest or longest path (T5). The highest comfort ratings, with 50% and 52%, respectively, were assigned to both map-related tasks where we asked participants to select polygons (T6 and T7), and the highest confidence ratings (70% and 59%) were assigned to the map-related tasks where we asked participants to select a line (T4) and to select a point (T2). In contrast, the low task success of T5 is also reflected by the low comfort and confidence ratings (20% and 23%, respectively).

**Figure 4.**
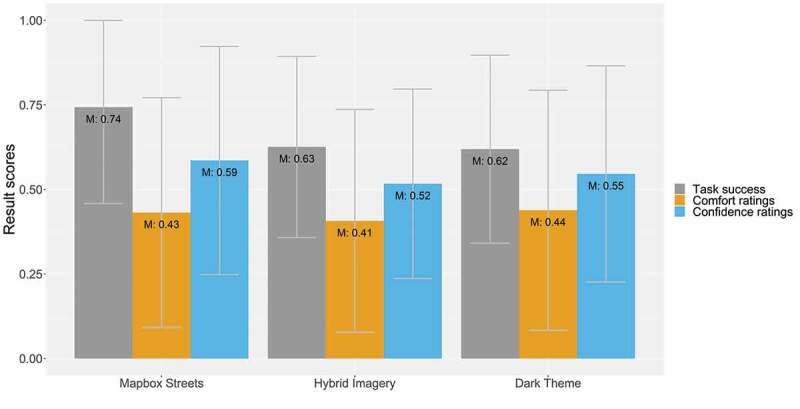
Task success, comfort, and confidence ratings by base map styles

Figure 4 shows the result scores of task success, comfort, and confidence ratings of each *base map style*. The Mapbox Streets base map yielded the highest task success and confidence ratings (74% and 59%). All other base map styles yielded similar result scores, with a task success of 63% and 62% for the hybrid imagery base map and dark theme, and comfort and confidence ratings ranging between 41% and 44% and 52% and 59%, respectively, for all three base map styles.

**Figure 5.**
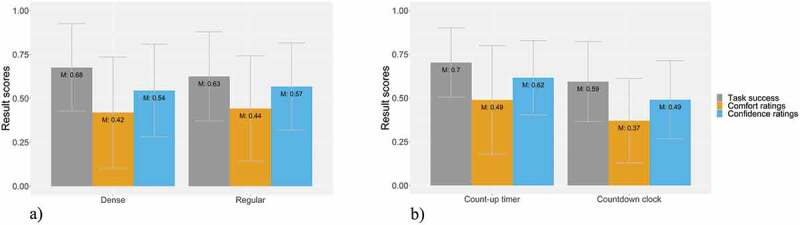
a) Task success, comfort, and confidence ratings by map detail densities; b) Task success, comfort, and confidence ratings by time pressure variants

Figure 5(a) shows the same result scores for the *map detail densities*. In general, there is no clear distinction between the task success, comfort, and confidence ratings between the two types of map detail densities.

Lastly, Figure 5(b) depicts result scores of the task success, comfort, and confidence ratings for the *time pressure* types. Naturally, all these result scores are higher for the count-up timer. For the countdown clock, the result scores are between 11 and 13 percentage points lower.

### 3.2. Task success, comfort, and confidence ratings by map elements

In addition to the overall task success, comfort, and confidence ratings of the participants, we were also interested in the result scores of each map element by map-related task.

**Figure 6.**
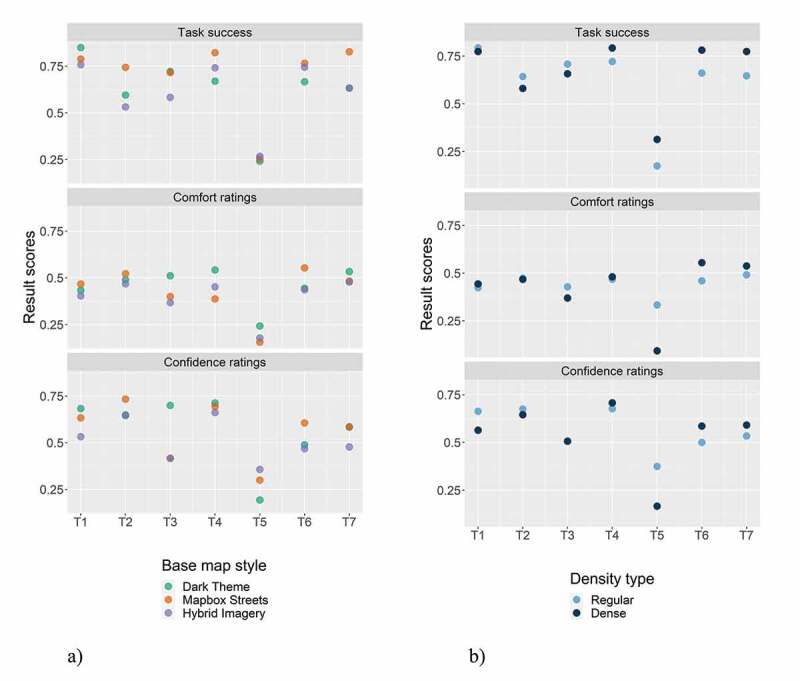
a) Task success, comfort, and confidence ratings of base map styles by map-related tasks; b) Task success, comfort, and confidence ratings of map detail densities by map-related tasks

Figure 6(a) shows the result scores for each base map style by map-related tasks. For most map-related tasks, the Mapbox Streets base map showed the highest task success. The most considerable difference between the Mapbox Streets base map and the other base maps was recorded for the map-related tasks of selecting a point (T2) and selecting detailed polygons (T7). The dark theme base map yielded a high task success for the map-related tasks of creating a point (T1) and selecting a point based on distance estimates (T3).

For the comfort and confidence ratings, again, Mapbox Streets and the dark theme base map yielded the highest scores. In particular, the Mapbox Streets base map scored high comfort and confidence ratings for the map-related tasks of creating a point (T1), selecting points (T2), and selecting generalized polygons (T6). In contrast, the dark theme base map yielded high result scores for the map-related tasks of selecting points based on distance estimates (T3) and selecting lines (T4).

Figure 6(b) depicts the result scores for each density type by map-related tasks. For the first three map-related tasks (where participants were asked to create a point, select points, and select points based on distance estimates; T1-3), the regular-density type (pre-defined filter rank by Mapbox) mostly yielded higher task success, comfort, and confidence ratings, but both density variations achieved very similar results. For the map-related tasks of selecting lines (T4), generalized polygons (T6), and detailed polygons (T7), the dense variant showed higher result scores. For the map-related task of selecting lines based on distance estimates (T5), the dense information variant yielded a higher task success, but the comfort and confidence ratings were higher for the regular-density information variant. This may be due to participants perceiving the map-related task as difficult, and a higher density of information further reduced the UX. For the map-related task of selecting lines (T4), both density variants scored very similar.

**Figure 7.**
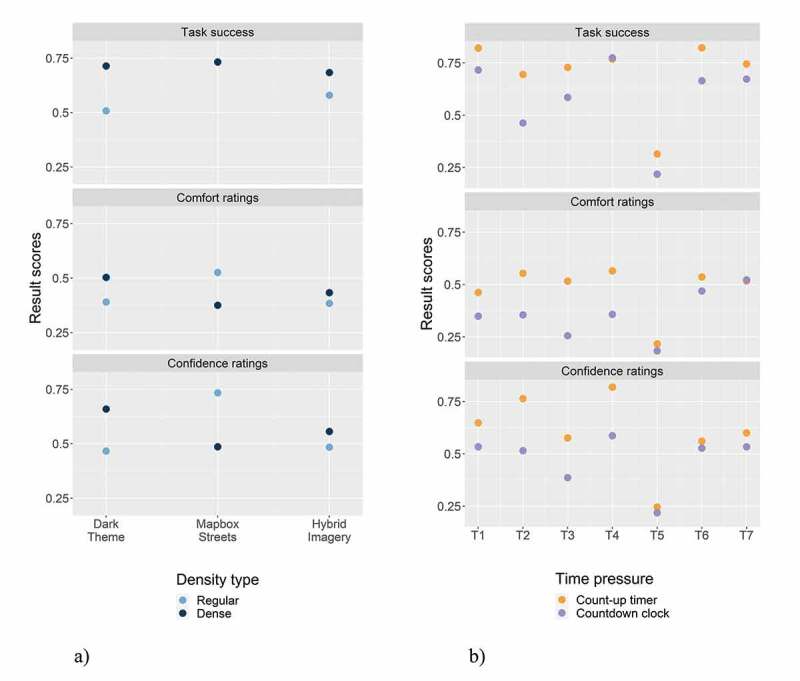
a) Task success, comfort, and confidence ratings of map detail densities by base map styles; b) Task success, comfort, and confidence ratings of time pressure variants by map-related tasks

In addition to the density type by map-related tasks, we were also interested in *how the density types performed with each base map* style (Figure 7(a)). While the Mapbox Streets base map yielded similar task success scores for both density variations, and higher comfort and confidence ratings for the regular-density variant, the dense variant performed better for the dark theme and hybrid imagery base maps for all result scores. In the dark theme base map, the visibility of POIs, street networks, or other relevant infrastructure is rather low because of the poor contrast between the base map and these features. Presumably, the more POIs are visible on this base map style, the easier it is for the participants to use. For the hybrid imagery base map, the dense variant performed slightly better, but the result scores are relatively similar for the two density variants; in particular, for the comfort and confidence ratings. Interestingly, the regular-density variant performed much better on the Mapbox Streets base map than with the other base map styles. Presumably, this comes down to the balance of readability and information visibility on this type of base map.

Lastly, Figure 7(b) visualizes the result scores for each *time pressure variant by map-related tasks*. In general, for most of the map-related tasks, the count-up timer yielded higher result scores. While we perceive this as a logical outcome, we aimed to evaluate how strong the differences are between the variants. The strongest difference between the time pressure variants is evident for all result scores for the map-related tasks of selecting points (T2) and selecting points based on distance estimates (T3). Regarding the comfort and confidence ratings, a similarly strong discrepancy is also evident for the map-related task of selecting lines (T4); though, the task success was similar for both density variants. In contrast, smaller differences between the variants are evident for both map-related tasks that involve selecting polygons (T6 and T7), as well as for the map-related task of selecting lines based on distance estimates (T5). However, the latter map-related task, in general, shows rather low result scores, which is why the difference between the variants is small.

### 3.3. Regression analysis

For the regression analysis, we built three models with three different dependent variables: task success, comfort, and confidence ratings of participants when solving map-related tasks (Table 5).

**Table 5.** Regression models with SBC and p-value with task success, comfort, and confidence ratings as dependent variables (with grayed-out areas indicating reference levels; ^1^ Regular-density as a reference level; ^2^ Count-up timer as a reference level; ^3^ Answer choice “no” as reference level)

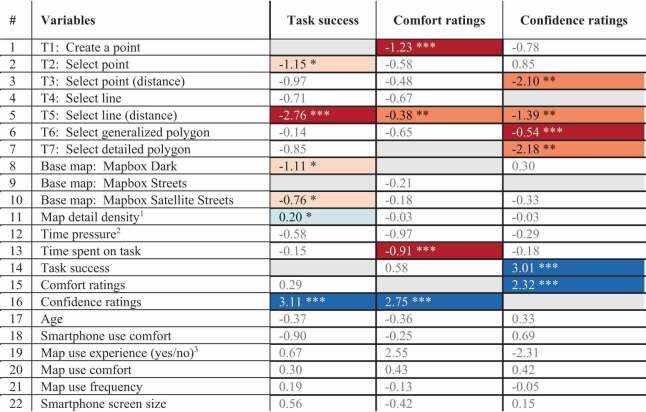

For the first regression model, with *task success* as the dependent variable, we used the map-related task of creating a point on the map (T1) as a reference level for the map-related task variants. With a significance level of p < 0.001 *** and p < 0.05 *, the map-related task of selecting lines based on distance estimates (T5) and the map-related task of selecting points (T2) are negatively correlated with task success. This is also supported by previously shown results (see Figure 3), where both map-related tasks exhibited the lowest task success of all map-related tasks. Regarding the base map styles, using the Mapbox Streets base map as a reference level, both other base map styles were negatively correlated with task success (p < 0.05 *). Also, with a significance of p < 0.05 *, higher map detail density correlated with higher success on map-related tasks. Participants’ confidence in their answers was further positively correlated with solving the map-related tasks correctly (p < 0.001 ***).

We built the second model with *participant comfort* as the dependent variable. We used the map-related task of selecting detailed polygons (T7) as a reference level for the map-related task category. The map-related tasks of creating a point (T1) and selecting lines based on distance estimates (T5) were significantly negatively correlated with participant comfort ratings (p < 0.001 *** and p < 0.01 **, respectively). The time spent on each task was another negatively correlated variable (p < 0.001 ***). Additionally, the participants who felt confident in solving the map-related tasks were also more likely to feel comfortable (p < 0.001 ***).

Lastly, the third model was built with *participant confidence* as the dependent variable. We used the map-related task of selecting lines (T4) as a reference level for the map-related task category. The map-related tasks of selecting points and lines based on distance estimates (T3 and T5) and the map-related tasks of selecting detailed and generalized polygons (T6 and T7) were all negatively correlated with participant confidence (p < 0.01 ** (T3, T5, and T7) and p < 0.001 *** (T6)). Further, participants that solved the map-related tasks correctly and felt comfortable when solving the map-related tasks were more likely to also feel confident in having solved the map-related tasks correctly (p < 0.001 ***).

In addition to running the regression analyses over the entire dataset, we were further interested in carrying out the regression analyses for each map element. However, we received a lower number of participants than expected due to the COVID-19 pandemic-related pivot in our study (see section 4 for further discussions on this topic). Therefore, as each map variation was used a fewer number of times than anticipated, we only ran the analysis for each map-related task and in support of the findings of the previous section 3.2. We found that the dark theme base map was negatively correlated with the task success of the map-related task to select lines (T4; p < 0.01 **). In addition, the dark theme and hybrid imagery base maps were both negatively correlated with the task success of the map-related task to select detailed polygons (T7; p < 0.05 *). For the confidence ratings, the hybrid imagery base map was negatively correlated with participants’ confidence in using the map-related task to create a point on the map (T1; p < 0.05 *); the Mapbox Streets and the hybrid imagery base maps were further both negatively correlated with participants’ confidence of using the map-related task to select points based on distance estimates (T3; p < 0.01 ** and p < 0.05 *, respectively). In terms of comfort ratings, we did not find any significant relationships between the map-related tasks and map elements. Annex Table A2, Table A3, and Table A4 (see supplementary materials) depict the full range of results of the regression models of each map-related task.

### 3.4. Preferences and general remarks

After each map variation, we asked the participants to provide feedback about perceived problems when completing the experiment. Table 6 summarizes these findings over all map variations. Since each of the 107 participants used twelve map variations, we collected responses to 1284 map variations. While participants indicated that 50% of the map variations were used without any problems, they also noted facing difficulties in finding the right information and zooming and panning on the map for 19% of the map variations. For a smaller fraction of the map variations (8%), participants perceived problems when trying to consult the popup information of the POIs. For 6% of the map variations, participants indicated difficulties with creating or selecting features on the map and problems with time constraints. For smaller fractions of the map variations (less than 6%), participants indicated difficulties with understanding the question text of the map-related tasks, too much information on the map, problems with colors and symbols, and toggling the layers.

**Table 6.** Perceived problems with map variations

Problem indication	Result scores
No problems	m = 0.5; n = 644
Find information	m = 0.19; n = 250
Zooming/panning	m = 0.19; n = 238
Popup	m = 0.08; n = 104
Create/select feature	m = 0.06; n = 83
Time constraint	m = 0.06; n = 81
Toggling layers	m = 0.05; n = 58
Question text	m = 0.04; n = 57
Information quantity	m = 0.04; n = 54
Colors	m = 0.04; n = 46
Symbols	m = 0.04; n = 46

In the last section of the survey, we also asked participants to provide feedback about their general base map preferences. 81% of our participants preferred the Mapbox Streets base map compared to the dark theme base map. When asked to choose between Mapbox Streets and the hybrid imagery base map, 78% of the participants again chose the Mapbox Streets base map. In contrast, 67% of the participants chose the hybrid imagery base map when asked to compare it to the dark theme base map.

We further asked the participants what they perceived as positive and negative regarding the maps and map-related tasks that we presented. Table 7 outlines the summaries of comments made by more than five participants.

**Table 7.** Positive and negative remarks as free-text feedback

Positive remarks	Negative remarks
Short, precise, and understandable question text (21 participants)	Moving the maps with two fingers (16 participants)
Icons and colors of the maps were comfortable and intuitive (13 participants)	Question text was not understandable (nine participants)
Interesting and diverse map-related tasks (twelve participants)	Dark theme base map was too dark (eight participants)
Clear maps with the right quantity of information (twelve participants)	Difficult to answer the questions (seven participants)
The possibility to toggle layers on and off (seven participants)	Time constraint (six participants)

## 4. Discussion and limitations

In our analysis of survey data, we were able to include the results of 107 participants who completed our survey, from a total of 205 participants that started the survey. 98 participants only registered their characteristics but did not complete all of the twelve map variations. As the application only registers the results of a completed survey section, containing all posed map variations, we do not have statistics on how many map variations participants completed before dropping out. However, this information could have helped in interpreting the reasons for the drop-out. Comparing the participants who only provided their basic information to those who completed the entire experiment, we found that the mean values of variables regarding smartphone use and map use experience were lower for participants that only registered than for those who completed the full survey. This could indicate that the survey was technically demanding and/or required too much time to complete (~20 minutes).

The aim of our study was to analyze the relation between selected context attributes (participant characteristics, map-related tasks, map design elements) and mobile map design element UX. We pursued this research question to examine whether the UX of mobile map elements may be modified by user context attributes. As in Vincent et al. (2019), Wilkening and Fabrikant (2011), and Biland and Çöltekin (2017), in addition to the task success, we also reported on self-rated comfort and confidence levels of the participants. In general, we found that participant comfort and confidence ratings were always lower than the task success. The difference between comfort/confidence and task success contradicts in the literature described observed tendencies of participants over-estimating their performance (Moore & Healy, 2008). As Biland and Çöltekin (2017) and Moore and Healy (2008) depict, participants’ self-reporting, and thus over- or under-estimating performance, depend on various reasons, such as expertise or gender. For our study, we particularly note differences between task success and comfort/confidence ratings between the map-related tasks, ranging from 0.07 and 0.12 percentage points for map-related tasks T2 and T5 (which performed worst in terms of task success) to 0.36 and 0.31 percentage points for map-related tasks T1 and T4 (which performed best in terms of task success). This is an interesting finding as map-related tasks that were solved more successfully show larger discrepancies to participants’ ratings than map-related tasks of low task success. These stronger differences were not found for the base map styles, map detail densities, and time pressure variants, though a decrease in task success led also to a decrease in the discrepancy between task success and participants’ ratings. Also, in addition to potentially other factors, such as gender or spatial literacy, we assume the lower self-reported comfort and confidence ratings may be because a) the participants were using the application for the first time and were not used to it, and b) the experimental setting with time pressure perhaps led to general discomfort.

Analyzing the specific reasons for the reported difference between task success and participants’ comfort and confidence is, however, out of scope of this article.

Ultimately, we did not find notable relationships between participant characteristics and map design UX. However, we found interesting clues to the interplay between map design choices and the different activities that users carried out with the maps (i.e. the map-related tasks). In the following sections, we discuss these findings regarding map-related tasks, base map choices, map detail densities, and time pressure variations.

**Map-Related Tasks**. The map-related task where participants were asked to select lines based on distance estimates (T5) yielded the poorest performance all around. In contrast, the map-related task where participants also were asked to select a line on the map but based on a specific attribute (T4) yielded the highest performance in terms of task success and comfort ratings. Hence, interacting with line features was easy for most participants, but choosing and selecting lines based on distance estimates (T5) was hard for our participants. 71% of these participants indicated having had problems, in particular with finding the POIs necessary to solve the map-related task. While 44% of the participants reported this difficulty on the dense map detail variant, only 25% reported this issue on the regular-density variant. Further, in common navigation map applications, users are guided in selecting the shortest or fastest routes by tools that make those choices automatically. For this map-related task, however, participants were *not provided with these tools*, which may be why their UX was poorly rated.

With respect to task success, selecting lines (T4) was as easy for participants as creating a point (T1). For the map-related tasks where participants were asked to select points on the map (T2 and T3), the result scores were relatively similar. However, the comfort and confidence ratings of the map-related task where participants had to select a point based on distance estimates (T3) were slightly lower compared to the map-related task of selecting points based on an attribute (T2), but the task success of the former (T3) was slightly higher. We assume that, as for T5, participants were less confident and comfortable in *visually interpreting distances*, as there was no guidance. This is also reflected in the remarks section of the participants, where 51% indicated having faced several problems (e.g. finding relevant POIs, moving the map to review POIs) in solving the map-related task T3. For T2, the participants were asked to interact with each point, read and interpret its annotation and icon, open its popup window to collect more information, and, finally, interpret all of this information to solve the map-related task. The depicted *additional interaction with the points might have led to a lower task success* than for the map-related task of estimating point distances (T3). However, participant performance was aided by coupling the map-related task T2 with a regular-dense Mapbox Streets base map.

For the map-related tasks of selecting polygons (T6 and T7), all result scores were similar between variants. Hence, we are *not able to detect differences in UX between generalized and more detailed and fragmented polygons*. In general, the task success was high compared to other map-related tasks, indicating participants’ general ease in working with polygons.

**Base Map Styles**. While the Mapbox Streets base map exhibits higher scores of task success compared to the other two base map styles, all base map styles are somewhat similar in their comfort and confidence ratings. In terms of comfort ratings, map-related tasks T3, T4, and T6 yielded the greatest difference between base map styles. Thereby, the dark theme base map performed best for the map-related tasks of selecting points based on distance estimates (T3) and selecting lines (T4). In contrast, the Mapbox Streets base map was rated best for the map-related task of selecting generalized polygons (T6). For the confidence ratings, again, the dark theme and Mapbox Streets base map performed best. A stronger discrepancy is evident for the map-related task of selecting points based on distance estimates (T3), whereby the dark theme base map yielded the highest ratings. General remarks of the participants indicated that *most of the participants preferred the Mapbox Streets base map and mentioned negative comments regarding the dark theme base map* (e.g. “the base map was too dark”). We found this discrepancy interesting, indicating that a dark theme base map was generally perceived as less useful. This was presumably because of the difficulty of reading information on it or because participants have not used this kind of base map previously, which, thereby, generated discomfort and led to an affective response by the participants. However, when the base map was coupled with map-related tasks where overlay information was more important (selecting points and lines – T2-T5), we assume that the base map helped to contrast and highlight these overlay features. In general, the hybrid imagery base map performed more poorly than the other two base maps, which is in concurrence with Konečný et al. (2011). However, for the comfort ratings, the scores were relatively similar to those of the other variants. The confidence ratings yielded the highest discrepancy between the base map variants, which indicates that participants were less sure about their task performance on the hybrid imagery base map.

**Map Detail Density**. While the *result scores were similar across the map detail densities*, statistically, participants were more likely to solve the map-related tasks on an information-dense map. This finding is supported by other studies, indicating that map detail complexity and its impact on location memory and learning performance follows an inverted U-shape (Bestgen et al., 2017; Keil et al., 2020; Liao et al., 2019; Lokka et al., 2018; Vincent et al., 2019). Neither low nor high levels of map detail complexities aid the users. Hence, our tested information-dense map provided an amount of map detail, which generally supported participants performance.

As Fairbairn (2006) and Liao et al. (2019) indicate, preferred map detail complexity may also be tied to the users’ abilities or the map-related tasks. In our study, for the map-related tasks of creating or selecting points (T1-T3), the comfort and confidence ratings were similar between the density variants, with a slight tendency toward higher scores for the regular-density map. For the map-related task of selecting lines (T4), the scores were almost the same between the variants. For the map-related task of selecting lines based on distance estimates (T5), participants were more successful with an information-dense variant but more comfortable and confident with a regular-density map. For both map-related tasks that involved selecting polygons (T6 and T7), the high-density map yielded better result scores.

In general, while the high-density variant performed better for the dark theme and hybrid imagery base maps, the Mapbox Streets base map worked better in combination with the regular-density variant. This may be a result of the superior readability of its base map content in contrast to the other two base map styles. Since Atri is a small village with only a few POIs visualized on the standard Mapbox base maps, even the dense map version was not cluttered with POIs. Still, we assume that a *lower quantity of base map details supported map-related tasks where participants had to interact more with overlay features* on the map (e.g. toggling layers, visually interpreting distances between POIs).

Hence, the overall design of the map and the map-related tasks is highly associated with the preferred map density variant (Liao et al., 2019). Nevertheless, the distinction between a regular-density and high-density map is rather vague when relying on the Mapbox POI filter rank and depends on the geographical area. An information-dense map in Atri, as a small village, might be very different than an information-dense map of a bigger city.

**Time Pressure**. In terms of time pressure, the map-related tasks employing a *countdown clock yielded lower result scores*, which supports reported findings by Wilkening and Fabrikant (2011). However, we further find that the effects of time pressure are also associated with the map-related tasks. For both map-related tasks that involved selecting polygons (T6 and T7), no clear distinction was evident between a count-up timer and a countdown clock, and there was only a slight difference for the map-related task of creating a point on the map (T1). The *greatest differences between the time pressure variants were observed for the map-related tasks of selecting points and lines* (T2-T4), where participants had to interact substantially with the overlay features (e.g. toggling layers, visually interpreting distances). For the map-related task of selecting lines based on distance estimates (T5), the result scores of both densities were similar due to the overall problems with this map-related task. In terms of the time spent on each map-related task, a greater response time for each map-related task significantly decreased the comfort of the participants. Again, this indicates that the overall map design is relevant for increasing or decreasing effects (in this case of time pressure) on the performance of the map user.

**Limitations**. Due to the COVID-19 pandemic, we had to substantially pivot our study to be able to carry out the experiment. More than 200 participants were initially expected to participate in a field survey in Atri, Italy, associated with a workshop series by the YouthMetre project, which was canceled due to the pandemic. Hence, the most important factors that impacted our study were the pivot to carrying out the experiment as an online survey and the decrease of our study participants. We expect that the pivot had a substantial impact on our experiment results, which we will reflect on in the following.

In our study analysis, we were able to detect differences in map design UX modified by the map-related tasks, but we were not able to detect a notable influence of participant characteristics on the UX. Even though the 84 map variations received a total of 1284 responses, each map variation was only answered 15 times due to the decrease of study participants. With more data for each map variation, it would have been possible to analyze the impact of participant characteristics on each map element on a deeper level, e.g. through regression analyses specifically for each map variation instead of over the entire dataset. Due to this issue, we only included the results of the regression models of the map-related tasks and in support of the findings of the descriptive statistics. However, for the most part, we are not able to detect significant relationships of the described differences between map-related tasks and map elements (e.g. base map styles). The described differences of section 3.2 should therefore be validated in future studies. We also have to note that the questions associated with each map-related task might have introduced additional errors as they might have been perceived differently by the participants. Further, while participants were given the option to indicate their level of comfort and confidence (e.g. feeling comfortable, indifferent, or uncomfortable) in the self-rating questions after each map variation, for simplification purposes, we opted to not further analyze the range of comfort and confidence. Additionally, as the survey was rather long (~20 minutes), potentially too technically demanding, and distributed as an online survey instead of the intended field survey, almost 50% of participants that started the survey quit before completing it. Reducing the length of the survey and testing only a few map variations might have helped retain participation until the end of the survey. With fewer testing conditions, it would then also be possible to opt for a within-subjects design and balance the conditions. Due to the large number of testing conditions in our study, we chose to apply a between-subjects design and randomized the conditions, which, however, might have introduced additional errors because of, e.g. individual differences between the participants.

Further, we may also need to consider how to acquire and analyze user context regarding map design UX in a different manner. In the present work, we did not consider environmental or temporally changing factors. These could constitute crucial context variables for typical work with mobile mapping tools. Additionally, there are many more variations in map design elements one could test in future studies.

## 5. Conclusion and future work

In this work, we evaluated mobile map UX to assess the interplay between user context attributes and mobile map design variations. We were particularly interested in how user context influences the UX of different map variations. As mobile mapping tools are potentially used for different purposes, users, and in different contexts of use, we sought to characterize the extent to which user context attributes might influence task success, comfort, and confidence ratings in relation to different map variations.

With this work, we aim to contribute to a growing body of literature assessing the impact of user context and user audience variety of map applications. In particular, this study extends the research assessing the relations between user context and the UX of mobile map elements. We, thereby, build on the approach by Griffin et al. (2017), who defined four components to understand a map use situation: user, environment, activity, and map. In this work, we evaluated the components of user (i.e. collected participant characteristics), activity (i.e. the map-related tasks), and map (i.e. the map design elements). In our findings, we see that the map-related tasks (activity) were the primary influencing factor on the map design UX.

The results of our study showed high task success, comfort, and confidence ratings of participants interacting with polygons on most of the map variations posed in this study. Participants were further able to interpret line features, e.g. as (segments of) streets, based on their relative location to other geographic features. Interactivity loaded map-related tasks (e.g. interpreting distances and popup information) with point and line features were completed more successfully when paired with base maps containing less information, thereby contrasting the overlay features (e.g. with a dark theme or Mapbox Streets base maps). In terms of base map styles, we detected an interesting controversy regarding the dark theme base map, as this base map was perceived with discomfort by the participants even though it provided a good contrast of overlay features. The hybrid imagery base map, in general, never performed substantially worse than the dark theme or Mapbox Streets base map. We, however, recommend designing maps carefully, which are using a hybrid imagery base map, as its association to other design elements and map-related tasks are influential on the users’ performance (i.e. accuracy). We further argue that – in circumstances where users are under time pressure – information-loaded maps and map-related tasks that require substantial interactions (e.g. reading popup information, toggling layers) are design choices that should be avoided to support the users.

As a result of our experiment, we emphasize the importance of continuing research on how user context impacts map design UX. Our results indicated that the activities a user might carry out on a map (i.e. map-related tasks) are critical drivers of the overall map design UX. In future work, we recommend focusing on additional map design elements to gain a deeper understanding of which context attributes may modify users’ UX. It is challenging to understand the full impact of user context and static as well as dynamic context attributes should be analyzed.

Deeper engagement may also be required over a sustained period of time in order to assess changes in UX vis-à-vis dynamic variations in contextual attributes. Dynamic and sustained UX evaluation methods may be useful to test variations of map design elements and relate them to a broader range of user context attributes. Dynamically logging interaction data may be one worthwhile approach (Hilbert & Redmiles, 2000; Savino et al., 2021). Developing new ways to dynamically evaluate map design UX would also support research on user context-based dynamic map design adaptations in a broader sense.

Future experiments with other user groups may augment our findings. In that regard, the quantity and type of map design variations should be reviewed, as it might be better to concentrate on specific elements and test these in more depth. It would also be interesting to review our findings and their transferability to other user context factors (e.g. map use situations in different environments and with different activities), which we did not include in this study.

## Supplementary Material

Supplemental Material

Supplemental Material

Supplemental Material

Supplemental Material

## Data Availability

Participants of this study did not agree for their data to be shared publicly, so supporting data is not available.
