# Supplementary material for: The role of user context in the design of mobile map applications
Source: Cartogr Geogr Inf Sci. 2021 Jul 6;48(5):432–48. doi: 10.1080/15230406.2021.1933595 (PMC8459706; doi:10.1080/15230406.2021.1933595)
Supplement: Supplemental Material [file TCAG_A_1933595_SM5603.docx]

| # | Map-related task: | Question: | Map element variation: |
| --- | --- | --- | --- |
| 1 | T1: Create point | Which place in the city do you think is the most interesting one for tourists? | Mapbox Dark + Regular |
| 2 |  | Which place would you choose to go for a coffee? | Mapbox Dark + Dense |
| 3 |  | Which place in the city do you think is the most interesting one for tourists? | Mapbox Streets + Regular |
| 4 |  | Atri has a few sport courts, can you identify one of them? | Mapbox Streets + Dense |
| 5 |  | Which place in the city do you think is the most interesting one for tourists? | Mapbox Satellite Streets + Regular |
| 6 |  | Which place in the city is one of the greenest? | Mapbox Satellite Streets + Dense |
| 7 | T2: Select point (attribute) | Which palazzo has something to do with a Roman cistern? | Mapbox Dark + Regular |
| 8 |  | The description of which church mentions a local artist? | Mapbox Dark + Dense |
| 9 |  | The description of which church mentions the year 1741? | Mapbox Streets + Regular |
| 10 |  | The description of which church mentions a Baroque style staircase? | Mapbox Streets + Dense |
| 11 |  | Which building has something to do with a Roman bath? | Mapbox Satellite Streets + Regular |
| 12 |  | Which description of a museum mentions the year 1912? | Mapbox Satellite Streets + Dense |
| 13 | T3: Select point (distance) | Which library is furthest away from the Chiesa Madonna del Rosario? | Mapbox Dark + Regular |
| 14 |  | Which is the supermarket furthest away from the Museo capitolare di Atri? | Mapbox Dark + Dense |
| 15 |  | Which is the library furthest away from the CONAD CITY supermarket? | Mapbox Streets + Regular |
| 16 |  | Which important place is closest to the supermarket Despar: Palazzo Duchi d'Acquaviva or Palazzo Mambelli? | Mapbox Streets + Dense |
| 17 |  | Which church is closest to the Auditorium Sant'Agostino? | Mapbox Satellite Streets + Regular |
| 18 |  | Which church is furthest away from the Parco Comunale? | Mapbox Satellite Streets + Dense |
| 19 | T4: Select line (attribute) | On which path can you pass two churches? | Mapbox Dark + Regular |
| 20 |  | On which path can you follow on which you pass by two educational instutions? | Mapbox Dark + Dense |
| 21 |  | Which route passes by a Palazzo? | Mapbox Streets + Regular |
| 22 |  | Which path passes by a museum and a palazzo? | Mapbox Streets + Dense |
| 23 |  | Which path can you walk where you pass by a church, a monastero and an important palazzo of Atri? | Mapbox Satellite Streets + Regular |
| 24 |  | Which path connects the Rocca di Capo D’Atri and Palazzo Mambelli? | Mapbox Satellite Streets + Dense |
| 25 | T5: Select line (distance) | A few streets are highlighted on the map: which one is the longest? | Mapbox Dark + Regular |
| 26 |  | Which path is the longest between the Parco Comunale and the Biblioteca del Centro (next to Palazzo Duchi d'Acquaviva? | Mapbox Dark + Dense |
| 27 |  | Which path is the longest between Chiesa San Nicola and Chiesa di Sant´Andrea Apostolo? | Mapbox Streets + Regular |
| 28 |  | Which path is the shortest one between Rocca di Capo D´Atri and Chiesa Di San Francesco? | Mapbox Streets + Dense |
| 29 |  | On which path do you have to walk the shortest distance between two churches? | Mapbox Satellite Streets + Regular |
| 30 |  | Which path between Rocca di Capo D´Atri and CONAD CITY supermarket is the shortest? | Mapbox Satellite Streets + Dense |
| 31 | T6: Select polygon (generalized) | Which area is best suited to represent the city outline? | Mapbox Dark + Regular |
| 32 |  | In which area can we find a theatre and a church? | Mapbox Dark + Dense |
| 33 |  | Which red-outlined area has the biggest overlap with zones of a high-density of places and landmarks? | Mapbox Streets + Regular |
| 34 |  | Which area best describes the location of the city with most of the relevant landmarks? | Mapbox Streets + Dense |
| 35 |  | Apart from the park and outside of the city– which area of the city is the greenest? | Mapbox Satellite Streets + Regular |
| 36 |  | Which of the areas has the greatest overlap with the Parco Comunale? | Mapbox Satellite Streets + Dense |
| 37 | T7: Select polygon | Which red-outlined area has the biggest overlap with zones of a high-density of places and landmarks? | Mapbox Dark + Regular |
| 38 |  | In which area do we find a palazzo and a church? | Mapbox Dark + Dense |
| 39 |  | In which area can we find a Palazzo and the Duomo Caffé? | Mapbox Streets + Regular |
| 40 |  | Which area best describes the location of the city with most of the relevant landmarks? | Mapbox Streets + Dense |
| 41 |  | Apart from the park and outside of the city– which area of the city is the greenest? | Mapbox Satellite Streets + Regular |
| 42 |  | In comparison – which is the red-outlined area that has the biggest overlap with green areas around the city? | Mapbox Satellite Streets + Dense |
