# Supplementary material for: The role of user context in the design of mobile map applications
Source: Cartogr Geogr Inf Sci. 2021 Jul 6;48(5):432–48. doi: 10.1080/15230406.2021.1933595 (PMC8459706; doi:10.1080/15230406.2021.1933595)
Supplement: Supplemental Material [file TCAG_A_1933595_SM5601.docx]

| **#** | Variables | T1: Create a point | T2: Select point | T3: Select point (distance) | T4: Select line | T5: Select line (distance) | T6: Select generalized polygon | T7: Select detailed polygon |
| --- | --- | --- | --- | --- | --- | --- | --- | --- |
| 1 | Base map: Mapbox Dark |  | -2.74 |  | -2.91 ** | 0.11 | -0.77 | -3.32 * |
| 2 | Base map: Mapbox Streets | -0.01 |  | 1.63 |  | -0.37 |  |  |
| 3 | Base map: Mapbox Satellite Streets | -2.23 | -3.23 | -0.01 | -0.02 |  | -0.56 | -1.65 * |
| 4 | Map detail density^1^ | 1.90 | -0.20 | -0.17 | 0.34 | 0.14 | 0.35 | 0.31 |
| 5 | Time pressure^2^ | -2.01 | -0.37 | -2.23 | 1.55 | 0.34 | -1.94 | -0.97 |
| 6 | Time spent on task | -0.75 | -0.03 | -0.29 | -0.57 * | 0.52 * | -1.24 * | 0.29 |
| 7 | Comfort ratings | -1.68 | 0.59 | 0.42 | 0.61 | -0.46 | 0.48 | -0.39 |
| 8 | Confidence ratings | 6.16 | 7.81 *** | 4.65 *** | 5.72 *** | 2.32 * | 1.72 ** | 3.01 ** |
| 9 | Age | -5.08 | -1.47 | 0.64 | -1.60 * | -1.72 * | 0.53 | -0.50 |
| 10 | Smartphone use comfort | -3.86 | -0.42 | 0.07 | -0.66 | 0.23 | -0.36 | -0.72 |
| 11 | Map use experience (yes/no)^3^ | 3.40 | -0.28 | 2.61 | -1.61 | -4.70 | 2.20 | 3.14 |
| 12 | Map use comfort | 3.14 | 0.22 | 0.11 | 0.30 | 0.33 | 0.69 | -0.09 |
| 13 | Map use frequency | -0.65 | 0.18 | 0.26 | 1.64 * | -1.11 | 0.31 | 1.02 |
| 14 | Smartphone screen size | 4.59 | 0.62 | 0.30 | -0.69 | 0.63 | 0.51 | 0.87 |
