# Supplementary material for: The role of user context in the design of mobile map applications
Source: Cartogr Geogr Inf Sci. 2021 Jul 6;48(5):432–48. doi: 10.1080/15230406.2021.1933595 (PMC8459706; doi:10.1080/15230406.2021.1933595)
Supplement: Supplemental Material [file TCAG_A_1933595_SM5600.docx]

| **#** | Variables | T1: Create a point | T2: Select point | T3: Select point (distance) | T4: Select line | T5: Select line (distance) | T6: Select generalized polygon | T7: Select detailed polygon |
| --- | --- | --- | --- | --- | --- | --- | --- | --- |
| 1 | Base map: Mapbox Dark | -0.37 | -0.51 |  |  |  | 0.20 |  |
| 2 | Base map: Mapbox Streets |  |  | 0.89 | -2.40 | -1.88 |  | -1.31 |
| 3 | Base map: Mapbox Satellite Streets | -0.46 | -0.47 | 0.35 | -0.22 | -1.24 | -0.05 | -0.47 |
| 4 | Map detail density^1^ | 0.14 | 0.13 | -0.09 | -0.16 | -0.35 | 0.21 | -0.04 |
| 5 | Time pressure^2^ | -1.46 | -2.21 | -2.33 * | -2.64 | -1.22 | -0.65 | 0.03 |
| 6 | Time spent on task | -1.21 | -1.88 * | 0.09 | -0.89 | -0.74 | -3.39 *** | -1.26 * |
| 7 | Task success | -0.31 | 1.81 | 0.45 | 3.55 | -3.69 | -0.68 | 0.49 |
| 8 | Confidence ratings | 2.05 *** | 2.30 ** | 2.68 *** | 4.48 *** | 2.87 *** | 2.53 *** | 2.83 *** |
| 9 | Age | 0.16 | -1.34 | 0.53 | -0.18 | 0.18 | -1.70 * | 0.11 |
| 10 | Smartphone use comfort | 0.90 | -1.01 | 0.14 | -0.52 | -0.35 | -0.37 | 0.36 |
| 11 | Map use experience (yes/no)^3^ | 3.83 | 1.68 | 2.22 | 34.84 | 3.67 | 2.60 | 25.63 |
| 12 | Map use comfort | 0.20 | 0.90 | -0.03 | 0.88 | 0.07 | 0.57 | 0.11 |
| 13 | Map use frequency | -0.14 | -0.88 | 0.81 | -1.49 | 0.48 | -0.10 | 0.53 |
| 14 | Smartphone screen size | 0.10 | -0.13 | -0.10 | 0.43 | -2.74 * | -0.48 | -1.58 |
