# Supplementary material for: The role of user context in the design of mobile map applications
Source: Cartogr Geogr Inf Sci. 2021 Jul 6;48(5):432–48. doi: 10.1080/15230406.2021.1933595 (PMC8459706; doi:10.1080/15230406.2021.1933595)
Supplement: Supplemental Material [file TCAG_A_1933595_SM5597.docx]

| **#** | Variables | T1: Create a point | T2: Select point | T3: Select point (distance) | T4: Select line | T5: Select line (distance) | T6: Select generalized polygon | T7: Select detailed polygon |
| --- | --- | --- | --- | --- | --- | --- | --- | --- |
| 1 | Base map: Mapbox Dark |  | 1.19 |  |  | -2.56 | -0.76 | 1.40 |
| 2 | Base map: Mapbox Streets | -0.76 |  | -3.24 ** | 0.48 | -0.26 |  |  |
| 3 | Base map: Mapbox Satellite Streets | -1.28 * | 1.37 | -1.38 * | -0.18 |  | -0.90 | -0.89 |
| 4 | Map detail density^1^ | -0.36 | 0.18 | 0.13 | -0.08 | -0.25 | 0.18 | -0.01 |
| 5 | Time pressure^2^ | -0.60 | -1.02 | -0.27 | -1.93 | 0.36 | 1.17 | -1.10 |
| 6 | Time spent on task | -0.41 | -0.32 | -0.27 | 1.45 ** | -0.02 | -0.20 | -0.89 |
| 7 | Task success | 1.37 | 12.01 *** | 3.27 * | 7.47 *** | 1.41 | 1.98 * | 2.11 * |
| 8 | Comfort ratings | 1.53 *** | 2.23 *** | 2.50 *** | 3.08 *** | 2.24 *** | 2.31 *** | 2.59 *** |
| 9 | Age | 0.33 | 0.82 | -1.02 | 3.08 *** | 0.15 | 0.24 | 0.35 |
| 10 | Smartphone use comfort | 0.40 | 0.57 | -0.25 | 1.36 * | -0.34 | -0.01 | 0.26 |
| 11 | Map use experience (yes/no)^3^ | -5.43 | 4.00 | -4.98 | 0.04 | -3.75 | 2.74 | -1.91 |
| 12 | Map use comfort | 0.26 | 1.32 | 0.54 | -0.60 | 0.27 | 1.58 | 0.07 |
| 13 | Map use frequency | 0.07 | -0.48 | -0.18 | 0.92 | 0.65 | -1.95 * | -0.17 |
| 14 | Smartphone screen size | 0.13 | -0.29 | -0.38 | 0.87 | 2.05 * | 0.17 | -0.29 |
